# Defective mitochondrial protein import contributes to complex I-induced mitochondrial dysfunction and neurodegeneration in Parkinson’s disease

**DOI:** 10.1038/s41419-018-1154-0

**Published:** 2018-11-07

**Authors:** Sandra Franco-Iborra, Thais Cuadros, Annabelle Parent, Jordi Romero-Gimenez, Miquel Vila, Celine Perier

**Affiliations:** 1 Neurodegenerative Diseases Research Group, Vall d’Hebron Research Institute-Center for Networked Biomedical Research on Neurodegenerative Diseases (CIBERNED)-Autonomous University of Barcelona, Barcelona, Spain; 2 Catalan Institution for Research and Advanced Studies (ICREA), Barcelona, Spain

## Abstract

Mitochondria are the prime energy source in most eukaryotic cells, but these highly dynamic organelles are also involved in a multitude of cellular events. Disruption of mitochondrial homeostasis and the subsequent mitochondrial dysfunction plays a key role in the pathophysiology of Parkinson’s disease (PD). Therefore, maintenance of mitochondrial integrity through different surveillance mechanisms is critical for neuronal survival. Here, we have studied the mitochondrial protein import system in in vitro and in vivo models of PD. Complex I inhibition, a characteristic pathological hallmark in PD, impaired mitochondrial protein import, which was associated with a downregulation of two key components of the system: translocase of the outer membrane 20 (TOM20) and translocase of the inner membrane 23 (TIM23), both in vitro and in vivo. In vitro, those changes were associated with OXPHOS protein downregulation, accumulation of aggregated proteins inside mitochondria and downregulation of mitochondrial chaperones. Most of these pathogenic changes, including mitochondrial dysfunction and dopaminergic cell death, were abrogated by TOM20 or TIM23 overexpression, in vitro. However, in vivo, while TOM20 overexpression exacerbated neurodegeneration in both substantia nigra (SN) pars compacta (pc) and striatum, overexpression of TIM23 partially protected dopaminergic neurons in the SNpc. These results highlight mitochondrial protein import dysfunction and the distinct role of two of their components in the pathogenesis of PD and suggest the need for future studies to further characterize mitochondrial protein import deficit in the context of PD.

## Introduction

Mitochondrial dysfunction has been implicated in many neurodegenerative diseases and in particular in Parkinson’s disease (PD)^1^. For the past 20 years, the bioenergetic deficit has been the most frequently recognized and well-characterized aspect of mitochondrial pathology in most PD models, with complex I inhibition being proved to be associated with dopaminergic cell death^2^. However, the new key questions are no longer whether a bioenergetic defect in mitochondria is sufficient to cause PD, but whether a failure in the mitochondrial quality control mechanisms is sufficient, or necessary, to cause PD.

Mitochondria contain approximately 1500 different proteins, 99% of which are encoded by the nuclear genome. Therefore, the import, sorting and assembly of nuclearly-encoded mitochondrial proteins is essential for normal mitochondrial function. Only 13 proteins of the respiratory chain are encoded by the mitochondrial genome and synthesized in the mitochondria^3^. Nuclearly-encoded mitochondrial proteins are synthesized in cytosolic ribosomes as precursor proteins and imported into mitochondria by evolutionarily conserved multi-subunit mitochondrial membrane translocases: translocase of the outer membrane (TOM) and translocase of the inner membrane (TIM)^4^. Precursor proteins with N-terminal presequences follow the presequence pathway and are imported through the TOM complex and the TIM23 complex^5^. Nearly all precursors initially enter mitochondria by passing a common entry gate formed by the TOM complex with the outer membrane receptor TOM20 acting as an initial docking site^6,7^. Presequence-containing precursor proteins are directly passed on from the TOM complex to the TIM23 complex without the need for soluble intermembrane space chaperones. TIM23 subunit of the TIM23 complex forms a Δψ-dependent protein-conducting pore across the inner membrane, enabling the translocation of matrix proteins and some inner membrane and intermembrane space proteins^8^. Complete import into the matrix additionally requires adenosine triphosphate (ATP) as a second energy source. Thus, nuclearly -encoded mitochondrial proteins use specific import systems for precise mitochondrial localization.

It is now clear that the targeting, import and assembly of mitochondrial proteins involve a complex series of events which are dependent on the mitochondrial translocation machinery and the information encoded in the imported protein^4,9^. Errors in these events can result in a protein not reaching its final destination, ultimately leading to a disease state in humans as is the case for the X-linked neurodegenerative disorder human deafness dystonia syndrome^10^ or for dilated cardiomyopathy with ataxia^11^. In addition to direct errors in the import process, mitochondrial protein import is a dynamic process that can be altered in response to various conditions, including oxidative stress, aging and external stimuli. Recent evidence indicates that α-synuclein, whose mutation/increased dosage/post-translational modification is associated with PD^12^, can translocate to mitochondrial membranes under pathogenic conditions, where it can cause complex I deficiency and increased production of reactive oxygen species (ROS)^13^. Previous studies have linked α-synuclein to the mitochondrial protein import machinery^14,15^. It was recently shown that certain species of α-synuclein bind specifically to TOM20, prevent its interaction with the co-receptor TOM22 and inhibit mitochondrial protein import^16^. However, it is an open question as to whether mitochondrial protein import alteration is associated with dopaminergic cell death and whether its restoration can attenuate mitochondrial dysfunction and dopaminergic cell death.

## Materials and methods

### Human post-mortem brain samples

Post-mortem human brain samples were obtained from the New York Brain Bank at Columbia University and the University of Barcelona Brain Bank according to the Vall d’Hebron Research Institute guidelines for sampling, including informed consent from the people involved or their representatives. Substantia nigra (SN) was dissected from ventral midbrain samples from 5 control subjects and 9 PD patients (mean age at death = 72 ± 4.3 years, cold post-mortem interval = 6.47 ± 2.72 h and frozen post-mortem interval = 10.63 ± 2.82 h). Total tissue proteins were isolated in a buffer containing 50 mM Tris-HCl (pH 7, Sigma-Aldrich, St. Louis, MO, USA), 150 mM NaCl (Sigma-Aldrich), 5 mM ethylenediaminetetraacetic acid (EDTA, Sigma-Aldrich), 1% sodium dodecyl sulfate (SDS, Sigma-Aldrich), 1% Nonidet P-40 (Sigma-Aldrich) and protease inhibitors (Complete mini Protease Inhibitor Cocktail, Sigma-Aldrich).

### Animals and treatments

All the animal procedures and methods employed in this study followed the Guide for the Care and Use of Laboratory Animals (Guide, 8th edition, 2011, NIH) and European (2010/63/UE), Spanish (RD53/2013) and Catalan (Decret 214/97) legislation. All the procedures had been approved by the Vall d’Hebron Research Institute Animal Ethical Experimental Committee. The 8–12-week-old C57BL/6Ncrl male mice received one intraperitoneal injection of MPTP-HCl per day (30 mg/kg of free base; Sigma-Aldrich) for 5 consecutive days and were euthanized at the indicated time points after the last 1-methyl-4-phenyl-1,2,3,6-tetrahydropyridine (MPTP) injection; control mice received saline injections only. Sample size was chosen according to previous experience in the lab and animals were allocated to different treatment groups by randomization.

### Viral vector production and stereotaxic delivery

For adeno-associated virus (AAV) delivery, human TIM23 and human TOM20 cDNA sequences cloned into a pCMV6-XL5 vector were obtained from Origene (Origene Technologies, Austin, TX, USA) and AAV of serotype 2/9 were produced at the UPV-CBATEG (Autonomous University of Barcelona, Spain). One microliter of viral suspension was stereotaxically delivered to the area just above the SN of 8-week-old C57BL/6Ncrl mice (−2.9 anterior-posterior, −1.3 medial-lateral and −4.2 dorsal-ventral) at a flow rate of 0.4 μl/min. At day 28 post AAV injections, animals were either euthanized or treated with saline or MPTP, as indicated.

### Tissue collection

For biochemical analysis, ventral midbrain was removed from the mice and resuspended in RIPA buffer. For histological analysis, mice were anesthetized with an intraperitoneal injection of 0.2 mL of 5% pentobarbital. We used a 9 mL/min flow rate of NaCl 0.9% (Frasenius Kabi, Bad Homburg, Germany) during 3 min and 4% paraformaldehyde (Panreac, Castellar del Vallès, Spain) diluted in 0.2 M phosphate buffer containing 0.15 M sodium phosphate dibasic and 0.05 M of sodium phosphate monobasic (Sigma-Aldrich) during 8 min. Brains were removed and incubated 24 h in 4% ice-cold paraformaldehyde at 4 °C and 48 h more in 30% sucrose (Sigma-Aldrich) at 4 °C. Brains were frozen in 2-methylbutane (Honeywell, Morris Plains, NJ, USA) between −30 and −40 °C and stored at −80 °C.

### Cell culture and treatments

Human neuroblastoma dopaminergic cell line BE(2)-M17 (Sigma-Aldrich, Ref. 95011816) was grown in Minimal Essential Medium optimized (Opti-MEM, Gibco, Thermo Fisher, Waltham, MA, USA) supplemented with 10% inactivated fetal bovine serum (Thermo Fisher), 5% penicillin–streptomycin (Sigma-Aldrich) and 0.5 mg/mL active geneticin (Thermo Fisher) and maintained at 37 °C in humidified 95% air/5% CO_2_ incubator. Transient transfection with complementary DNAs (cDNAs) was performed with Lipofectamine 2000 (Thermo Fisher), following manufacturer's recommendations. MitoGFP plasmid was graciously given by Dr. B. Dehay. The plasmid was produced using the mitochondrial-targeting sequence of Aconitase 2 and cloned into the pcDNA3 vector. Human TIM23 and TOM20 plasmids cloned into a pCMV6-XL5 vector were obtained from Origene (Origene Technologies), respectively. For drug treatments, cells were grown to 70–80% confluence and treated for 24 and 48 h. The 1-methyl-4-phenylpyridinium (MPP^+^, Sigma-Aldrich) was diluted in water and added at the following concentrations: 0.25, 0.5, 1, 2 and 5 mM. Samples were allocated to different treatment groups by randomization and researchers were blinded to the group assignment when performing the experiments and until the data were ready for statistical analysis.

### Quantitative real-time PCR (RT-qPCR)

RNeasy Mini Kit (Qiagen, Hilden, Germany) was used for messenger RNA (mRNA) extraction according to manufacturer’s instructions. Then, 1 μg of total mRNA was reverse-transcribed with Oligo(dT) using SuperScript III™ first-strand system for RT-PCR (Invitrogen, Carlsbad, CA, USA) or the High Capacity cDNA Reverse Transcription Kit (Applied Biosystems, Carlsbad, CA, USA) in a final volume of 20 μL following manufacturer’s instructions. Gene expression was analyzed using Taqman Universal Master Mix II with UNG (Roche Applied Biosystems) and fluorescence-labeled specific probes (Life Technologies, Carlsbad, CA, USA) for *CLPX* (#Hs01101010_m1), *HSP9* (#Hs00269818_m1) and *RPLP0* (#4326314E) on a 7900HT SDS (Applied Biosystems, CA, USA). Then, 2.5 ng of cDNA was used to measure mRNA levels. Relative quantification was carried out using the 2-deltadeltaCt method using the software ABI PRISM 7900HT SDS version 2.2 (Applied Biosystems).

### Mitochondrial DNA copy number

Purification of total DNA from cells and ventral midbrain tissue was performed using the QIAamp DNA Mini Kit (Qiagen) according to manufacturer’s instructions. To quantify mitochondrial DNA (mtDNA) content, relative mtDNA (12S rRNA gene, for cells; 16S rRNA and ND4 genes, for mouse tissue) was analyzed versus nuclear DNA (RNaseP gene, for cells; ANG1 gene, for mouse tissue) copy number. A standard curve with cloned amplicons was used for absolute quantification of mtDNA and nuclear DNA in the samples. Real-time PCR was performed on an ABI PRISM 7900HT and SDS version 2.2 software using Taqman Universal Master Mix II with UNG.

### Immunofluorescence, immunohistochemistry and quantitative morphology in mouse tissue

For immunohistochemistry and immunofluorescence studies, SN and striatum coronal sections were cut at either 20 μm or 30 μm using a cryostat (Leica, Wetzlar, Germany). Double immunofluorescence was performed in free-floating sections with a monoclonal antibody against tyrosine hydroxylase (TH) (1:1000; Chemicon, Waltham, MA, USA, MAB 5280), COX IV (1:500; Abcam, Cambridge, MA, USA, ab16056), Tim23 (1:1000; BD Biosciences, Bedford, MA, USA, 611222) or Tom20 (1:1000; Abcam, ab56783). Nuclei were visualized using Hoechst 33342 (1:20,000, Invitrogen) and images were acquired using an Olympus BX61 fluorescence microscope connected to an Olympus DP70 camera and cellSens software (Olympus Corporation). To assess Tim23 and Tom20 expression in free-floating SN sections, we used Vector® M.O.M™ Immunodetection kit (Vector Laboratories, Burlingame, CA, USA) according to manufacturer’s instructions and primary antibodies anti-Tim23 (1/300, Abcam) and anti-Tom20 (1/300, Abcam). To assess TH expression in SN, free-floating sections were incubated with polyclonal anti-TH (1:2000 for SN and 1:5000 for striatum; Calbiochem, Burlington, MA, USA, 657012). To develop we used the 3,3’-diaminobenzidine tetrahydrochloride (DAB Enhanced, Sigma-Aldrich). The total number of TH-positive SNpc neurons was estimated by stereology using the optical fractionator method (StereoInvestigator; MBF Bioscience, Williston, VT) in a Zeiss Axio Imager D1 microscope (Carl Zeiss Microscopy, Oberkochen, Germany) and the extent of striatal dopaminergic denervation was determined by optical densitometry using Sigma Scan software (Systate Software, Inc.). Immunohistochemistry and stereological cell counts were performed in a blind manner with respect to the treatment administered.

### Immunofluorescence in cells

Cells were fixed in 4% paraformaldehyde and immunostained with Ndusf3 (1:200; Abcam, ab110246), COX IV (1:300; Abcam), Tim23 (1:300; BD Biosciences) and Tom20 (1:300; Abcam). Cell nuclei were visualized using Hoechst 33342 at 10 μM for 10 min and mitochondria were labeled using MitoTracker™ Deep Red FM (Molecular Probes, Eugene, OR, USA). Fluorescence was analyzed using a Zeiss Axio Imager D1 fluorescence microscope or an Olympus FV1000 confocal microscopy (Tokyo, Japan). Quantifications were performed using ImageJ 1.50a (National Institutes of Health, USA).

### Immunoblot

Human, mouse and cellular protein extracts were homogenized in RIPA buffer and incubated in the following primary antibodies: mouse anti-Tim23 (1:1000, BD Biosciences), mouse anti-Tom20 (1:1000, Abcam), mouse anti-Total OXPHOS (1:1000, Abcam, ab110411), rabbit anti-eGFP (1:1000, Novus Biologicals, Littleton, CO, USA, NBP2-37821), rabbit anti-ClpX (1:2000, Abcam, ab168338), mouse anti-Grp75 (1:1000, Abcam, ab2799), mouse anti-Ndufs3 (1:1000, Abcam), rabbit anti-COX IV (1:1000, Abcam), rabbit anti-VDAC1/Porin (1:2000, Abcam, ab15895), mouse anti-α-Tubulin (1:5000, Sigma-Aldrich, T5168) and mouse anti-β-actin (1:5000, Sigma-Aldrich, A5441).

### Mitochondrial protein aggregation

Mitochondria were isolated as previously described in Frezza et al^17^. For assessing the presence of insoluble proteins in mitochondria, isolated mitochondria were resuspended at a final concentration of 1 mg/mL in lysis buffer containing 25 mM Tris-HCl (pH 7.4), 300 mM NaCl, 5 mM EDTA, proteases inhibitors and 1 mM phenylmethylsulfonyl fluoride. An equal volume of lysis buffer containing 1% Nonidet P-40 was added to mitochondrial dilution. Samples were incubated on ice during 10 min and centrifuged at 20,000 × *g* for 30 min at 4 °C. We collected both the supernatant, which is the soluble fraction, and the pellet, which represents the NP-40-insoluble aggregate. The insoluble fraction was centrifuged again at 20,000 × *g* for 15 min at 4 °C. The pellet was resuspended in loading buffer 4× containing blue 4× (100 mM Tris, 30% glycerol, 4% SDS and 0.2% bromophenol blue, Sigma-Aldrich) and 25 mM dithiothreitol in running buffer and both soluble and insoluble fractions were separated on 15% SDS-PAGE gels for western blot analysis using Total OXPHOS antibody. This protocol was adapted from Moisoi et al^18^.

### Mitochondrial pOTC import assay

Human ornithine transcarbamylase (OTC) precursor cDNA in pGEM-3Zf(+)-pOTC plasmid was transcribed and translated in vitro using the TNT-coupled reticulocyte lysate system (Promega) in the presence of l-[35S]methionine (PerkinElmer, Waltham, MA, USA). Following translation, [35S]methionine-labeled pOTC was incubated with isolated mitochondria at 37 °C for the indicated times, and mitochondria containing imported OTC were collected by centrifugation at 9000 × *g* for 10 min and subjected to SDS-PAGE. The radioactive polypeptides were visualized on the gel using a Personal Molecular Imager FX (Bio-Rad). Cleaved mature OTC (mOTC), which represents the completion of import into the mitochondrial matrix and migrates faster than precursor OTC (pOTC) on SDS-PAGE, was quantified using ImageJ 1.50. Data are presented as the percentage of mOTC compared to total [35S]pOTC.

### Mitochondrial membrane potential

Cells were washed twice with pre-warmed phosphate-buffered saline (PBS) and tetramethylrhodamine methyl ester (TMRM) probe (reconstituted in dimethyl sulfoxide (DMSO), Life Technologies) was added to the medium at 1 μM and incubated for 30 min in a cell incubator. Using a FACSAria flow cytometer (BD Biosciences, NJ, USA), 10,000 cells were acquired and fluorescence was read at 561 nm laser with 582/15 emission filter. Membrane potential was analyzed as mean fluorescence intensity and represented as fold change compared to the corresponding controls using the FCS Express (v3 De Novo SoftwareTM).

### ROS production

Cells were washed twice with pre-warmed PBS and 10 μM CM-H_2_DCFDA probe (reconstituted in DMSO, Life Technologies) was diluted in Opti-MEM medium and incubated for 30 min in a cell incubator. Cells were lysed using a cell lysis buffer and 50 μL of cell lysate was loaded per triplicate into a 96-well black microplate (Corning Inc., Corning, NY, USA) and read immediately with 485/20 nm excitation and 528/20 nm emission wavelengths in the spectrofluorometer (FLx800, Biotek Instruments, Inc., VT, USA). Fluorescence values were corrected for the protein concentration and represented as fold change compared to the corresponding controls.

### Cell death

Cell death was analyzed using the MTT Cell Proliferation Assay (ATCC bioproducts, Manassas, VA, USA) following manufacturer’s instructions and also as the number of propidium iodide (PI, Invitrogen)-positive cells. Using a FACSAria flow cytometer, 10,000 cells were acquired and fluorescence was read at 488 nm laser with 610/20 emission filter. Cell death was analyzed as the number of PI-positive cells and represented as fold change compared to the corresponding controls using the FCS Express (v3 De Novo SoftwareTM).

### Statistical analysis

The values were expressed as the mean ± s.e.m. The significant differences (*P < 0.05, **P < 0.01, ***P < 0.001) when comparing two groups were determined by a two-tailed unpaired Student’s *t*-test. When comparing more than two groups, significant differences were determined by one-way or two-way analysis of variance (ANOVA) followed by Tukey’s post hoc test. Statistical analyses were performed using GraphPad Prism (version 6.0, GraphPad Software Inc., San Diego, CA, USA).

## Results

### Mitochondrial protein import machinery deficiency in substantia nigra from PD brains

**Fig. 1:**
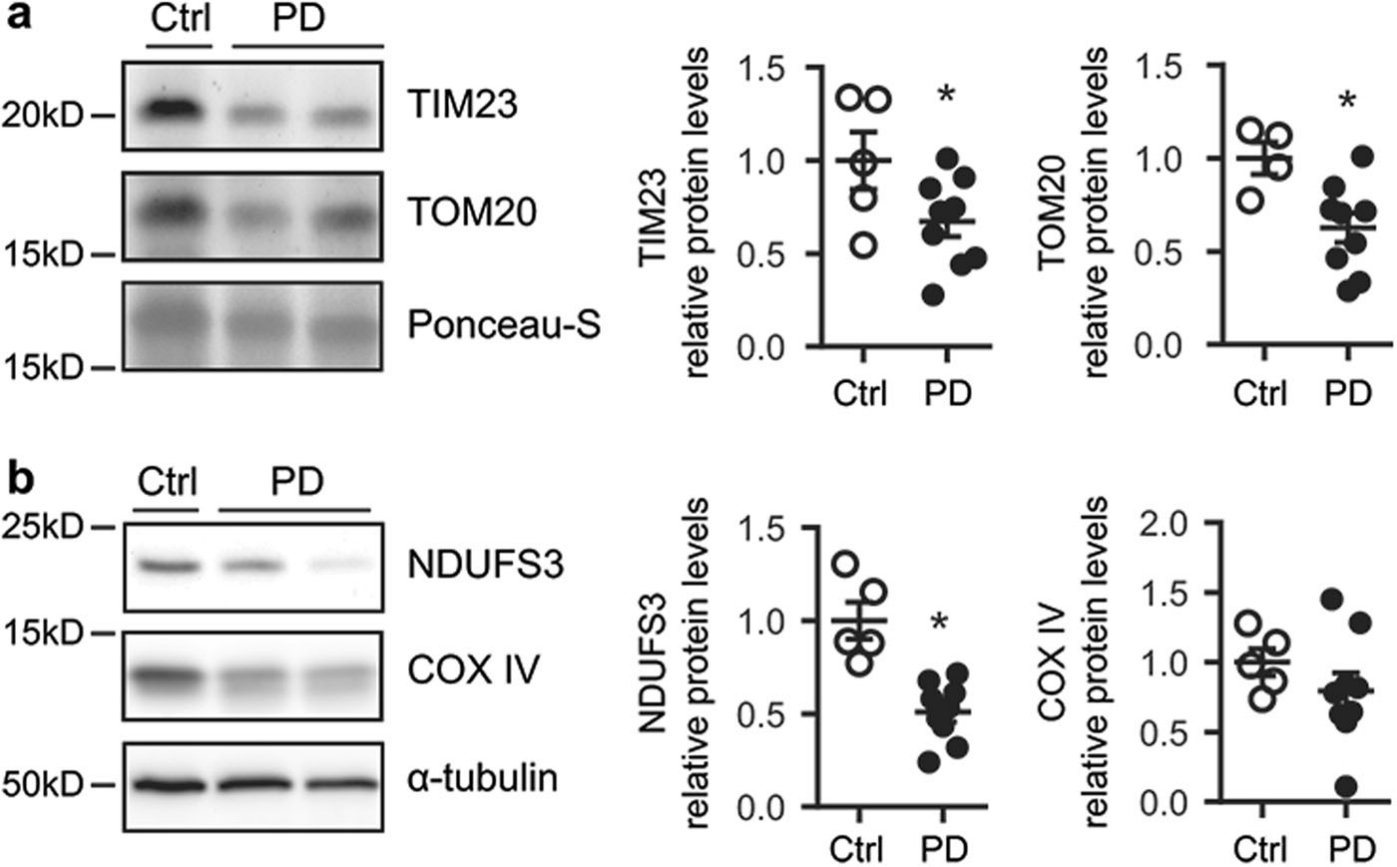
Mitochondrial protein import machinery deficiency in human substantia nigra in PD. **a** Representative immunoblots of TIM23 and TOM20 protein levels in substantia nigra homogenates from Ctrl (*n* = 4–5) and PD patients (*n* = 9). Protein levels were normalized relative to Ponceau-S. Quantification is depicted as fold change to Ctrl (*P < 0.05 after unpaired two-sided Student’s t-test). **b**) Representative immunoblots of NDUFS3, COX IV and α-tubulin protein levels in substantia nigra homogenates from control (Ctrl, *n* = 5) and PD patients (*n* = 9). Protein levels were normalized relative to α-tubulin (*P < 0.05 after unpaired two-sided Student’s *t*-test). Error bars indicate s.e.m.

To study the mitochondrial protein import machinery in the context of PD, we analyzed the expression of the mitochondrial translocases TIM23, located in the inner membrane, and TOM20, located in the outer membrane, in post-mortem samples from PD patients and control individuals. TIM23 and TOM20 protein levels were decreased in SN protein homogenates from PD patients compared to age-matched controls (Fig. 1a). To further determine whether mitochondrial protein import machinery was impaired in the SN of PD patients, we analyzed the expression levels of two nuclear-encoded mitochondrial proteins, NADH (reduced form of nicotinamide adenine dinucleotide)–ubiquinone oxidoreductase core subunit S3 (NDUFS3) and cytochrome *c* oxidase subunit IV (COX IV) by immunoblotting. NDUFS3 protein levels were decreased in SN protein homogenates from PD patients compared to age-matched controls, while COX IV protein levels had a tendency to decrease, though not significant (Fig. 1b). All together, these results could indicate the presence of an impaired mitochondrial protein import in PD patients. However, since post-mortem samples offer and end-point image, other PD-related mechanisms might be also taking place.

### Complex I inhibition impairs the import of proteins into mitochondria

**Fig. 2:**
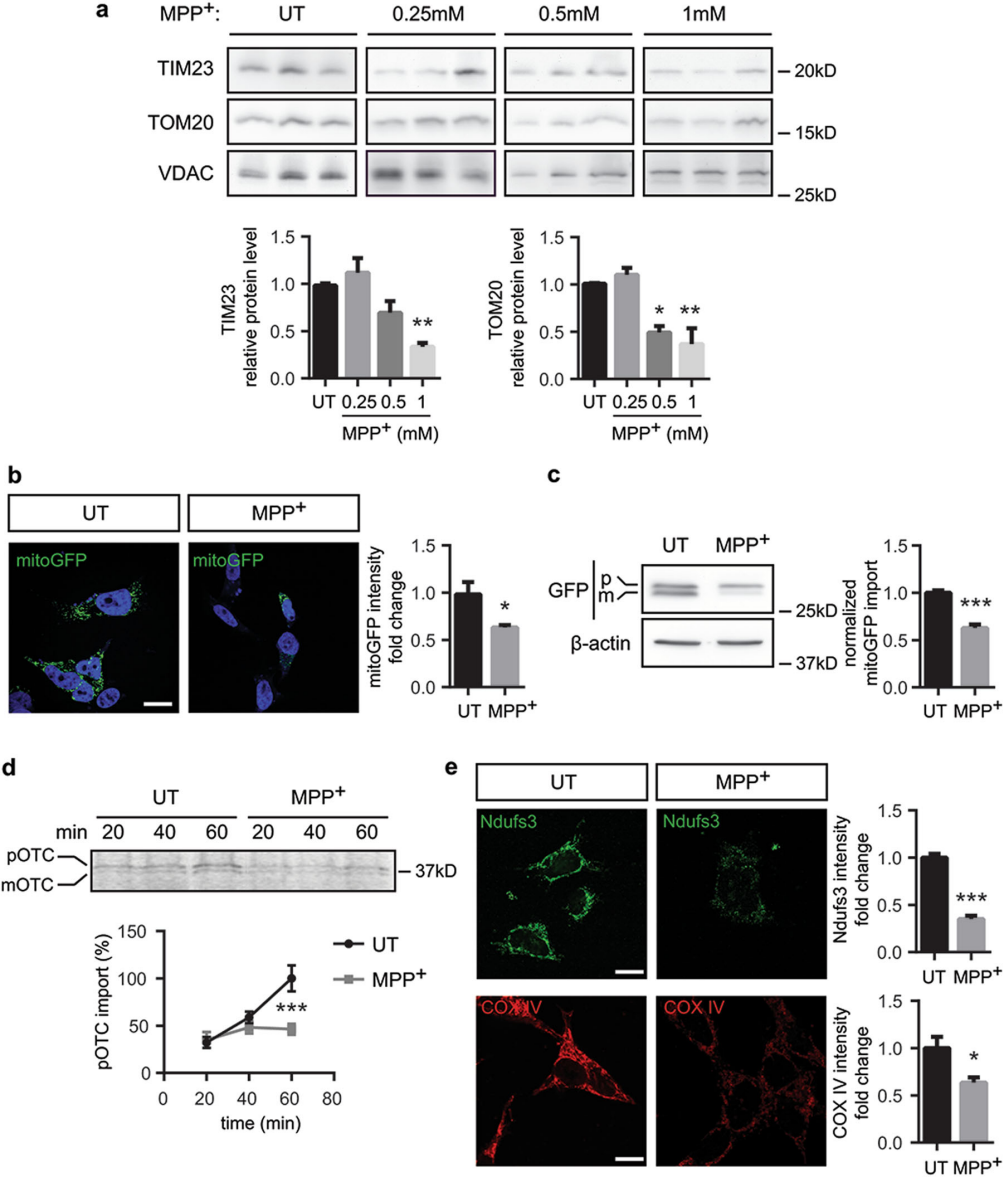
Complex I inhibition impairs mitochondrial protein import activity. **a–c**, **e** BE(2)-M17 cells were treated with MPP^+^ (0.25, 0.5 or 1 mM as stated) or untreated (UT) for 24 h. **a** Representative immunoblots of TIM23, TOM20 and VDAC protein levels normalized relative to VDAC (*n* = 3 independent experiments). Quantification is depicted as fold change to UT condition (*P < 0.05, **P < 0.01 after one-way ANOVA test followed by Tukey’s post hoc test). **b** Representative images of mitoGFP-transfected BE(2)-M17 cells. Quantification is depicted as the fold change of mitoGFP intensity per cell compared with UT condition. A minimum of 35 cells were analyzed per condition (*P < 0.05 after unpaired two-sided Student’s *t*-test). Scale bar: 10 μm. **c** Representative immunoblots of GFP and β-actin protein levels. mitoGFP import was calculated as the ratio of mature (m) GFP compared to the sum of precursor (p) and mature GFP (total GFP; *n* = 5 independent experiments, ***P < 0.001 after unpaired two-sided Student’s *t*-test). **d** Representative immunoblots of radiolabelled OTC in isolated mitochondria UT or treated with MPP^+^ (1 mM) at different time points. pOTC import was calculated as the percentage of mature (m) OTC compared to the sum of precursor (p) and mature OTC (*n* = 3 independent experiments, ***P < 0.001 after unpaired two-sided Mann–Whitney test). **e** Representative images of NDUFS3 and COX IV immunofluorescence. Quantification is represented as the fold change in NDUFS3 or COX IV intensity compared with UT condition. A minimum of 35 cells were analyzed per condition (*P < 0.05, **P < 0.01 after unpaired two-sided Student’s *t*-test or unpaired two-sided Mann–Whitney test). Scale bar: 10 μm. Error bars indicate s.e.m.

The data obtained in human post-mortem tissue suggest that mitochondrial protein import dysfunction might be associated with PD. Yet, human post-mortem studies can hardly provide mechanistic clues for such hypothesis. Therefore, we analyzed mitochondrial protein import in the dopaminergic neuroblastoma cell line BE(2)-M17 treated with MPP^+^, the active metabolite of parkinsonian neurotoxin MPTP, which inhibits complex I. Similarly to human post-mortem tissue, TIM23 and TOM20 protein levels were decreased in a dose-dependent manner in cells treated with MPP^+^ (Fig. 2a). To study whether downregulation of mitochondrial translocases correlates with decreases in mitochondrial protein import, we used the following methods previously reported in Di Maio et al:^16^ (i) confocal imaging and immunoblotting of mitochondrially targeted green fluorescent protein (mitoGFP), (ii) direct assay of mitochondrial protein import in isolated mitochondria and (iii) confocal measurements of mitochondrial localization of endogenous, nuclear-encoded, and imported proteins NDUFS3 and COX IV. Initial characterization was performed using mitoGFP fusion protein, in which the mitochondrial-targeting sequence has to be cleaved inside the mitochondria to release the mature fluorescent protein. MitoGFP intensity levels appeared decreased in the mitochondria of transfected cells following MPP^+^ intoxication (Fig. 2b). Processing of mitoGFP inside mitochondria leads to a slight drop in its molecular weight, thus making the precursor and the mature protein distinguishable by immunoblotting. Decreased mitoGFP fluorescence intensity in MPP^+^-intoxicated cells corresponded to a reduction in the mitoGFP intra-mitochondrial processing by ~35% (Fig. 2c), suggesting that mitochondrial import of proteins containing a mitochondrial-targeting sequence is impaired upon complex I inhibition. To address whether MPP^+^ directly inhibits mitochondrial protein import machinery, we isolated mitochondria and assessed mitochondrial protein import in organello using radio-labeled human OTC^19,20^. In this system, MPP^+^ intoxication inhibited OTC import by 50% as observed by the reduction in the mature OTC form (Fig. 2d). Finally, we examined the levels of endogenous nuclear-encoded, mitochondrially targeted NDUFS3 and COX IV, and found a decrease in both imported proteins after MPP^+^ treatment (Fig. 2e). Moreover, mitochondrial mass, reflected by mtDNA copy number, did not change in MPP^+^-treated cells (Supplementary Figure S1), suggesting that the downregulation of the parameters analyzed is not caused by a diminution of mitochondrial mass, but rather due to an intrinsic defect inside each mitochondrion.

**Fig. 3:**
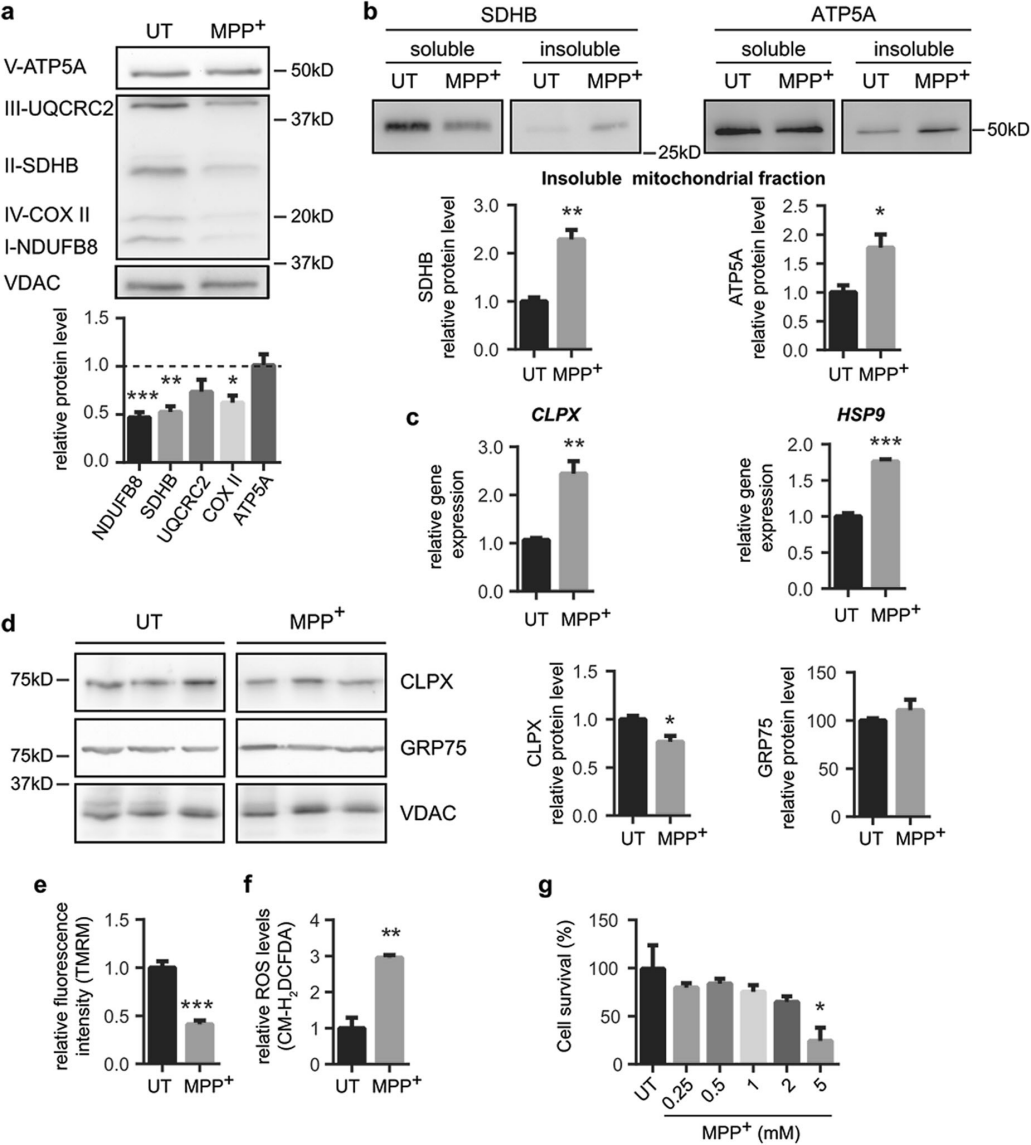
Complex I inhibition leads to a mitochondrial dysfunction and cell death. **a**–**f** BE(2)-M17 cells were treated with MPP^+^ (1 mM, 24 h) or untreated (UT). **a** Representative immunoblots of ATP5A (complex V), UQCRC2 (complex III), SDHB (complex II), COX II (complex IV), NDUFB8 (complex I) and VDAC protein levels normalized relative to VDAC (*n* = 3 independent experiments). Quantification is depicted as fold change to UT condition (*P < 0.05, **P < 0.01, ***P < 0.001 compared with UT condition after unpaired two-sided Student’s *t*-test). **b** Representative immunoblots of SDHB and ATP5A protein levels in soluble and insoluble mitochondria-isolated fractions (*n* = 3 independent experiments). Quantification is depicted as fold change to UT condition (*P < 0.05, **P < 0.01 after unpaired two-sided Student’s *t*-test). **c** *CLPX* and *HSP9* gene expression levels normalized relative to *RPLP0* (*n* = 3 independent experiments). Quantification is depicted as fold change to UT condition (**P < 0.01, ***P < 0.001 after unpaired two-sided Student’s *t*-test). **d** Representative immunoblots of CLPX, GRP75 and VDAC protein levels normalized relative to VDAC (*n* = 4 independent experiments). Quantification is depicted as fold change to UT condition (*P < 0.05 after unpaired two-sided Student’s *t*-test). **e** Mitochondrial membrane potential measured as TMRM fluorescence intensity. Quantification is depicted as the fold change in fluorescence intensity compared with UT condition (*n* = 3 independent experiments, ***P < 0.001 after unpaired two-sided Student’s *t*-test). **f** ROS production measured as CM-H_2_DCFDA fluorescence intensity. Quantification is depicted as the fold change in fluorescence intensity compared with UT condition (*n* = 3 independent experiments, **P < 0.01 after unpaired two-sided Student’s *t*-test). **g** In vitro sensitivity of BE(2)-M17 cells to increasing MPP^+^ concentrations for 24 h (*n* = 4 independent experiments). Cell survival was determined by MTT assay. Quantification is depicted as the % of cell survival relative to UT condition (*P < 0.05 after one-way ANOVA test followed by Tukey’s post hoc test). Error bars indicate s.e.m.

To determine the consequences of mitochondrial protein import impairment in vitro following MPP^+^ intoxication, we first assessed the protein levels of various OXPHOS subunits, as the majority of them are encoded by both the nuclear and the mitochondrial genomes. We analyzed NADH:ubiquinone oxidoreductase subunit B8 (NDUFB8, complex I), succinate dehydrogenase complex iron sulfur subunit B (SDHB, complex II), ubiquinol-cytochrome *c* reductase core protein 2 (UQCRC2, complex III) and ATP synthase F1 subunit alpha (ATP5A, complex V), which are nuclear-encoded, while cytochrome *c* oxidase subunit II (COX II, complex IV) is encoded by the mtDNA^21^. MPP^+^ intoxication led to a downregulation of all the proteins, except ATP5A (Fig. 3a). Moreover, we observed that MPP^+^-treated cells exhibited an enrichment of SDHB and ATP5A mitochondrial proteins in detergent-insoluble mitochondrial fractions (Fig. 3b), indicating an increase of aggregated intra-mitochondrial proteins. Interestingly, MPP^+^-treated cells exhibited a transcriptional activation of mitochondrial chaperones ATP-dependent Clp protease ATP-binding subunit ClpX-like (CLPX) and heat shock protein 9 (HSP9) (Fig. 3c), which was not observed at protein levels (Fig. 3d), suggesting that import defects might impair the mitochondrial translocation of chaperones and the activation of the mitochondrial unfolded protein response (mtUPR)^22^. One of the main functional consequences of complex I inhibition is the generation of ROS together with the loss of mitochondrial membrane potential^23,24^. Indeed, MPP^+^-dependent complex I inhibition led to increased ROS production and loss in mitochondrial membrane potential (Fig. 3e). MPP^+^-induced cell death occurred at the highest dose of MPP^+^ used (5 mM, Fig. 3f). These results indicate that mitochondrial protein import deficiency, intra-mitochondrial protein aggregation and mitochondrial dysfunction occur in an in vitro paradigm of PD, below the toxic threshold (5 mM MPP^+^), thus suggesting that these occur prior to cell death (Fig. 3f). These findings set the stage to study whether restoration of mitochondrial protein import machinery can attenuate mitochondrial dysfunction and cell loss in this pathological situation.

### TOM20 overexpression restores mitochondrial dysfunction and prevents cell death in vitro

**Fig. 4:**
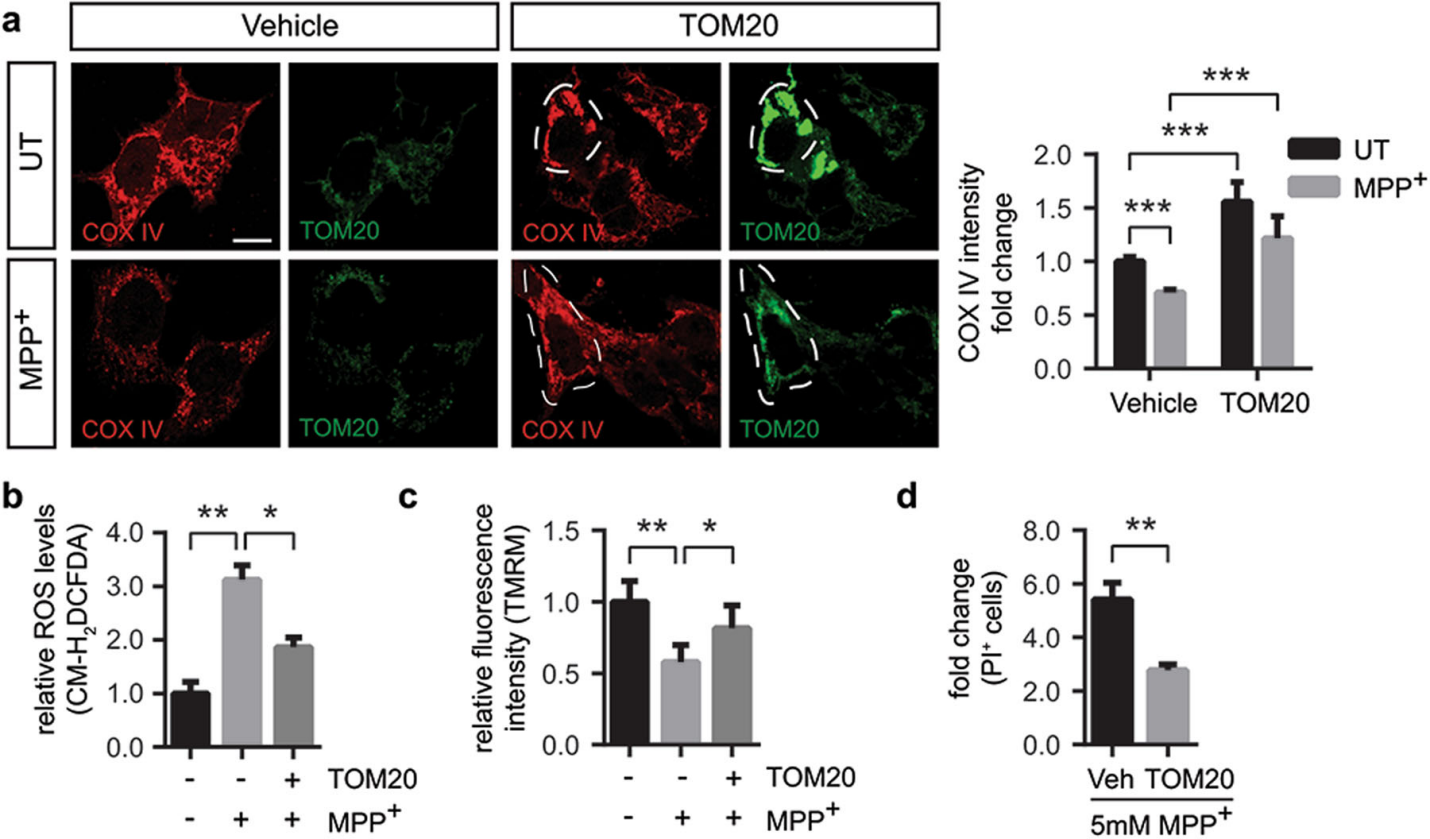
TOM20 overexpression restores mitochondrial dysfunction and prevents cell death in vitro. Vehicle- or pCMV6-XL5-TOM20-transfected (TOM20) BE(2)-M17 cells were untreated (UT) or treated with **a**–**c** 1 mM MPP^+^ for 24 h or **d** 5 mM for 24 h. **a** Representative images of COX IV and TOM20 immunostained cells. Quantification is depicted as the fold change in COX IV intensity compared with vehicle-transfected UT condition. Dashed line shows TOM20-overexpressing cells. A minimum of 15 cells were analyzed per condition (***P < 0.001 after two-way ANOVA followed by Tukey’s post hoc test). Scale bar: 10 μm. **b** ROS production measured as CM-H_2_DCFDA fluorescence intensity. Quantification is depicted as the fold change in fluorescence intensity compared with vehicle-transfected UT condition (*n* = 3 independent experiments, *P < 0.05, **P < 0.01 after one-way ANOVA followed by Tukey’s post hoc test). **c** Mitochondrial membrane potential measured as TMRM fluorescence intensity. Quantification is depicted as the fold change in fluorescence intensity compared with vehicle-transfected UT condition (*n* = 5 independent experiments, *P < 0.05, **P < 0.01 after one-way ANOVA followed by Tukey’s post hoc test). **d** Cell death measured as propidium iodide (PI)-positive cells (*n* = 5 independent experiments). Quantification is depicted as the fold change in the percentage of PI-positive cells compared with each control condition (**P < 0.01 after unpaired two-sided Student’s *t*-test). Error bars indicate s.e.m.

Since MPP^+^-induced mitochondrial protein import machinery deficiency is associated with cell death, we next determined whether restoration of key mitochondrial protein import translocases’ protein levels can attenuate MPP^+^-induced dysfunction. TOM20 belongs to the TOM complex in the outer mitochondrial membrane, where it acts as a receptor^25,26^ and has been previously reported to interact with α-synuclein^16^. Therefore, we first analyzed whether transient overexpression of pCMV6-XL5-TOM20 (TOM20) could prevent the impairment of the import system. Transient transfection led to significant increases in protein levels and a mitochondrial distribution of TOM20 (Supplementary Figure S2a and S2b). To analyze mitochondrial protein import system we focused on COX IV immunofluorescence levels, since COX IV is not directly inhibited by MPP^+^. Upon MPP^+^ intoxication there was a ~40% decrease in COX IV immunofluorescence intensity (Fig. 4a) which was prevented by TOM20 overexpression (Fig. 4a), indicating that MPP^+^-induced impairment in COX IV mitochondrial levels was reverted. Next, we examined ROS levels and mitochondrial membrane potential upon TOM20 overexpression. While complex I inhibition resulted in a threefold increase in ROS levels, TOM20 overexpression reduced ~33% ROS production after MPP^+^ intoxication (Fig. 4b). Mitochondrial membrane potential was also affected by MPP^+^ intoxication, whereas TOM20 overexpression partially rescued mitochondrial membrane potential to control levels (Fig. 4c). Since ROS production and loss of mitochondrial membrane potential are factors that precede mitochondrial-induced cell death, we also analyzed whether TOM20 overexpression protected against complex I-induced cell death. As shown before, the 5 mM dose of MPP^+^ induced a massive cell death in BE(2)-M17 cells (Fig. 3f). TOM20 overexpression exerted a partial protection against MPP^+^-induced cell death (Fig. 4d). All in all, TOM20 overexpression protected against complex I-induced mitochondrial dysfunction and cell death in vitro but only partially, indicating that other mechanisms beyond protein import contribute to the mitochondrial demise observed after MPP^+^ intoxication.

### TIM23 overexpression prevents cell death in vitro

**Fig. 5:**
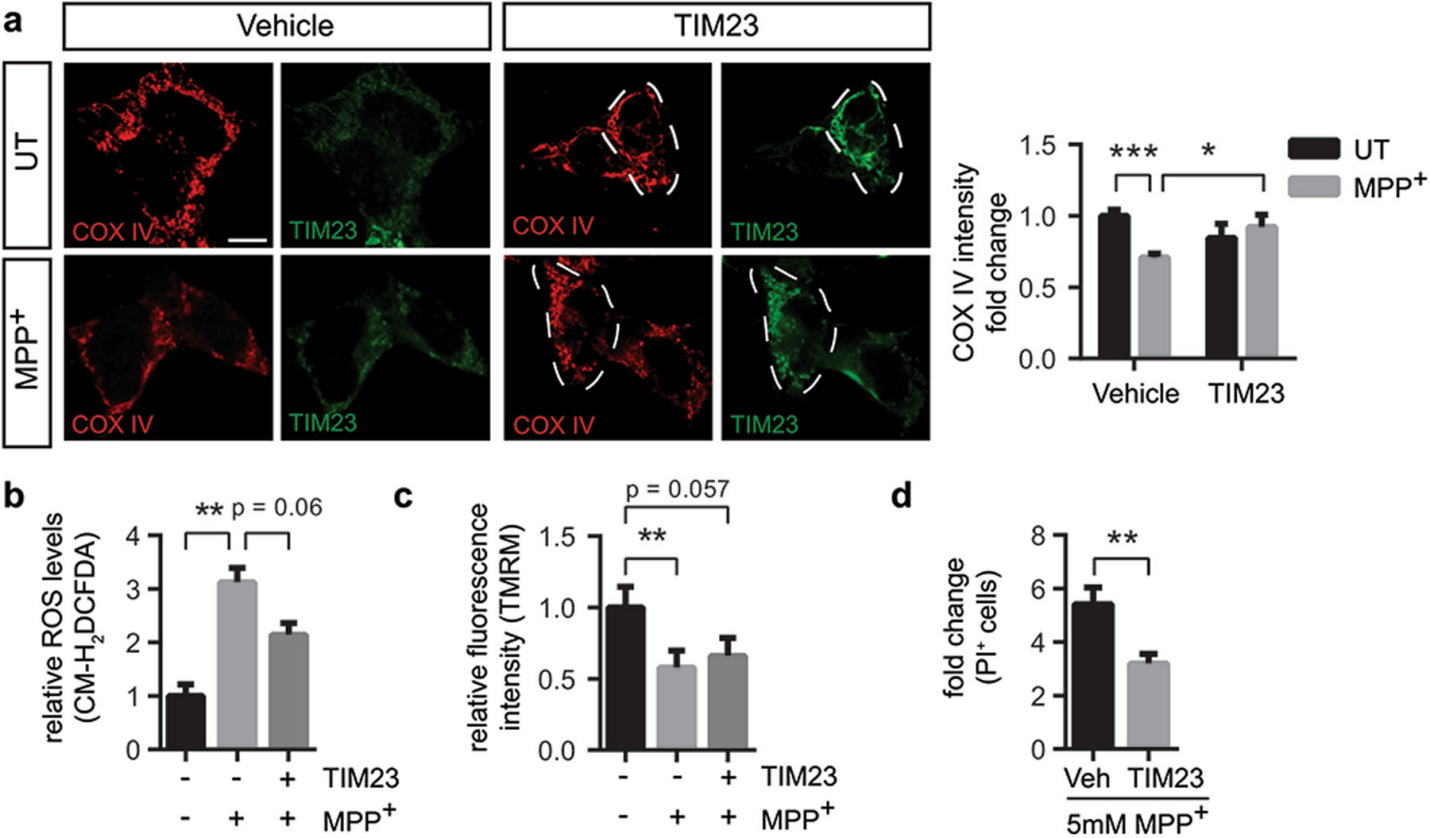
TIM23 overexpression restores mitochondrial dysfunction and prevents cell death in vitro. Vehicle- or pCMV6-XL5-TIM23-transfected (TIM23) BE(2)-M17 cells were untreated (UT) or treated with **a**–**c** 1 mM MPP^+^ for 24 h or **d** 5 mM for 24 h. **a** Representative images of COX IV and TIM23 immunofluorescence. Quantification is depicted as the fold change in COX IV intensity compared with vehicle-transfected UT condition. Dashed line shows TIM23-overexpressing cells. In all, 15 to 60 cells were analyzed per condition (*P < 0.05, ***P < 0.001 after two-way ANOVA followed by Tukey’s post hoc test). Scale bar: 10 μm. **b** ROS production measured as CM-H_2_DCFDA fluorescence intensity. Quantification is depicted as the fold change in fluorescence intensity compared with vehicle-transfected UT condition (*n* = 3 independent experiments, **P < 0.01 after one-way ANOVA followed by Tukey’s post hoc test). **c** Mitochondrial membrane potential measured as TMRM fluorescence intensity. Quantification is depicted as the fold change in fluorescence intensity compared with vehicle-transfected UT condition (*n* = 5 independent experiments, **P < 0.01 after one-way ANOVA followed by Tukey’s post hoc test). **d** Cell death measured as propidium iodide (PI)-positive cells (*n* = 5 independent experiments). Quantification is depicted as the fold change in the percentage of PI-positive cells compared with each control condition (**P < 0.01 after unpaired two-sided Student’s *t*-test). Error bars indicate s.e.m.

We hypothesized that decreased levels of TIM23 following complex I inhibition could also play an important role in mitochondrial dysfunction. Therefore, overexpression of TIM23 would potentially lead to increased presence of inner mitochondrial membrane channels, enabling the transport of precursor proteins into the matrix^27^. Transient expression of pCMV6-XL5-TIM23 (TIM23) led to a significant mitochondrial overexpression of TIM23 (Supplementary Figure S3a and S3b) and prevented the decrease in COX IV immunofluorescence intensity induced by MPP^+^ (Fig. 5a), suggesting that TIM23 alone can also have an influence in the translocation of mitochondrial proteins. Although TIM23 overexpression in MPP^+^-intoxicated cells barely decreased ROS production (Fig. 5b) and did not rescue mitochondrial membrane potential (Fig. 5c), TIM23 overexpression partially protected against MPP^+^-induced cell death (Fig. 5d). These results show that overexpression of TIM23 without rescuing neither ROS nor membrane potential is sufficient to mitigate complex I-dependent cell death.

### Mitochondrial protein import machinery impairment precedes dopaminergic neuron degeneration in the MPTP mouse model of Parkinson’s disease

**Fig. 6:**
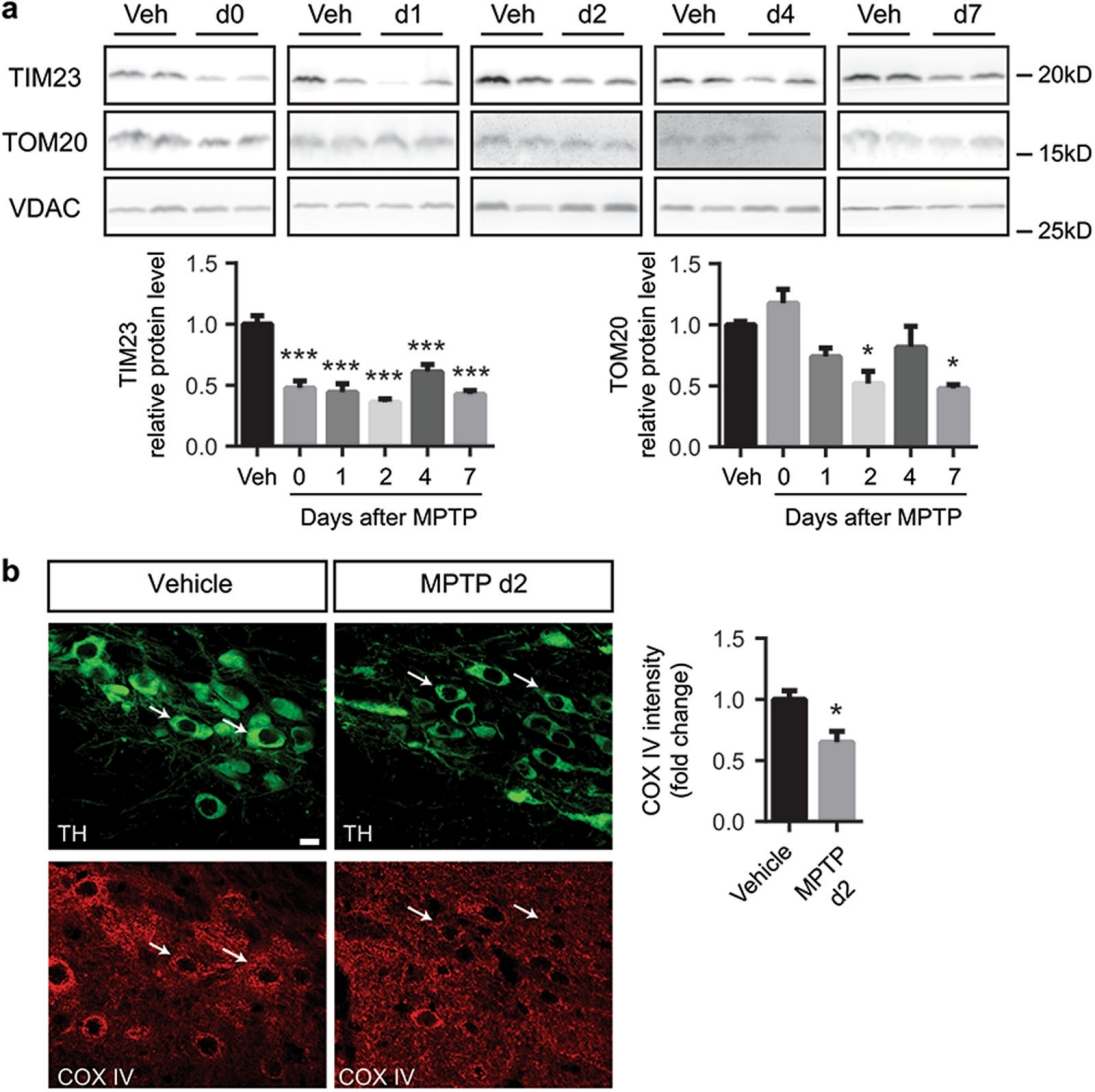
Mitochondrial translocases loss precedes dopaminergic neuron degeneration upon MPTP intoxication. **a** Representative immunoblots of TIM23, TOM20 and VDAC protein levels in ventral midbrain of vehicle- (Veh, *n* = 5) and MPTP-treated (*n* = 6–7) mice euthanized at different time points. Protein levels were normalized relative to VDAC and quantification is depicted as fold change to vehicle condition (*P < 0.05, ***P < 0.001 after one-way ANOVA followed by Tukey’s post hoc test). **b** Representative images of tyrosine hydroxylase (TH) and COX IV immunostained in ventral midbrain sections from vehicle- (*n* = 5) or MPTP-treated (*n* = 5) mice euthanized at day 2 after the last injection. Quantification is depicted as the fold change of COX IV intensity in TH-positive neurons compared to vehicle condition. White arrows indicate representative neurons. A minimum of 36 neurons were analyzed per animal (*P < 0.05 after unpaired two-sided Student’s *t*-test). Error bars indicate s.e.m.

Our in vitro data suggest that TIM23 and TOM20 deficiency are involved in dopaminergic neurodegeneration. To determine the relevance of these mitochondrial protein import proteins in an in vivo situation, we assessed TIM23 and TOM20 protein levels in the MPTP mouse model of PD, one of the most used mouse models to study mitochondrial dysfunction and cell death in the context of PD^24,28–33^. Here we found that MPTP (30 mg/kg/day for 5 consecutive days) induced a decrease of TIM23 protein levels in the ventral midbrain of intoxicated mice as early as at day 0 post MPTP injection (Fig. 6a), preceding dopaminergic cell death in this model, which occurs between days 2 and 4 post MPTP^24,32,33^. The deficit of TOM20 protein levels started at day 2, at the same time as dopaminergic cell death (Fig. 6a). Immunohistochemistry of TIM23 and TOM20 revealed a specific decrease within SN pars compacta (pc) dopaminergic neurons in these animals as early as 2 days after MPTP intoxication (Supplementary Figure S4) without any changes in mitochondrial mass (Supplementary Figure S5), suggesting a specific downregulation of TIM23 and TOM20 translocases. Ventral midbrain sections of MPTP-intoxicated mice euthanized at day 2 post MPTP (prior to the initiation of cell death in this model) were used to analyze COX IV immunofluorescence levels. MPTP intoxication was associated with decreased intensity of mitochondrially localized COX IV in TH-positive neurons of the SN (Fig. 6b). Since mitochondrial mass is not affected at this time point, these results suggest that COX IV immunofluorescence decrease could be caused by an impairment in the mitochondrial protein import machinery in SNpc dopaminergic neurons following MPTP intoxication. COX IV downregulation occurs within a time-frame compatible to influence the fate of dopaminergic neurons in this pathological situation.

### Modulation of mitochondrial protein import machinery in the MPTP mouse model of Parkinson’s disease

**Fig. 7:**
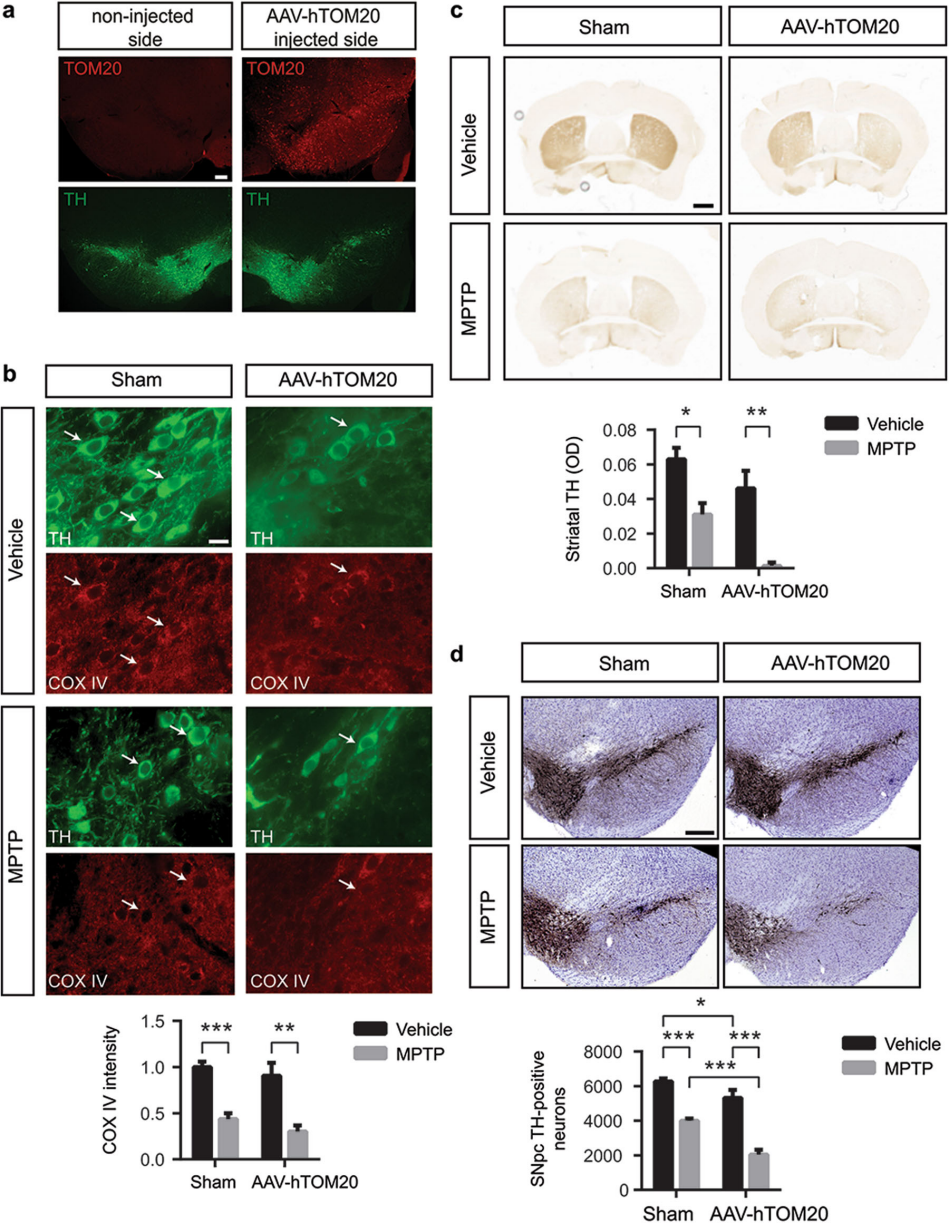
TOM20 overexpression cannot attenuate MPTP-induced dopaminergic neuron injury in vivo. **a** Representative images of TOM20 and TH immunofluorescence in AAV-hTOM20 mice. Scale bar: 150 μm. **b**–**d** Sham- and AAV-hTOM20-injected mice were treated with vehicle or MPTP. **b** Representative images of TH and COX IV immunofluorescence in ventral midbrain sections (Sham-Vehicle *n* = 5; Sham-MPTP *n* = 6; AAV-hTOM20-Vehicle *n* = 4; AAV-hTOM20-MPTP *n* = 4). Quantification is depicted as the fold change in COX IV intensity compared with sham-injected vehicle-treated group (**P < 0.01, ***P < 0.001 after two-way ANOVA followed by Tukey’s post hoc test). Scale bar: 50 μm. **c** Representative photomicrographs of TH immunohistochemistry in striatum (Sham-Vehicle *n* = 7; Sham-MPTP *n* = 5; AAV-hTOM20-Vehicle *n* = 8; AAV-hTOM20-MPTP *n* = 7). Quantification is depicted as the optical densitometry of striatal TH immunoreactivity in the different experimental groups at day 21 post MPTP (*P < 0.05, **P < 0.01 after two-way ANOVA followed by Tukey’s post hoc test). Scale bar: 500 μm. **d** Representative photomicrographs of TH immunohistochemistry in SNpc (Sham-Vehicle *n* = 14; Sham-MPTP *n* = 10; AAV-hTOM20-Vehicle *n* = 7; AAV-hTOM20-MPTP *n* = 7). Quantification is depicted as the stereological cell counts of SNpc TH-immunoreactive neurons in the different experimental groups at day 21 post MPTP (*P < 0.05, ***P < 0.001 after two-way ANOVA followed by Tukey’s post hoc test). Scale bar: 500 μm. Error bars indicate s.e.m.

Our in vitro and in vivo results associate defects in mitochondrial protein import with dopaminergic cell death. Moreover, both TOM20 and TIM23 induced a partial protection against complex I-induced cell death in vitro. Therefore, we analyzed whether restoration of TOM20 and TIM23 protein levels in vivo has an instrumental role in MPTP-induced neurodegeneration. Mice received single unilateral stereotaxic injections of vehicle or AAV-TOM20 in the SNpc. Sham and TOM20-overexpressing mice were treated with either saline or MPTP and we assessed (i) the mitochondrial protein import system by COX IV immunofluorescence, and (ii) the integrity of the nigrostriatal dopaminergic system at day 21 after the last MPTP injection, once the dopaminergic lesion is stabilized^33^. At 4 weeks after viral vector delivery, ventral midbrain samples ipsilateral to AAV-TOM20 injections displayed a 63% ± 4.81 transduction efficiency (Fig. 7a). MPTP intoxication induced a reduction in mitochondrial protein import as determined by the decrease in the mitochondrially localized COX IV protein (Fig. 7b). In contrast to in vitro results, TOM20 overexpression did not reverse the mitochondrial protein import deficit induced by MPTP (Fig. 7b). Regarding the integrity of the dopaminergic system, MPTP produced an ~50% depletion of striatal dopaminergic terminals in sham-injected mice, as assessed by optical densitometry of striatal TH-positive fibers (Fig. 7c). However, TOM20 overexpression did not prevent striatal terminal loss caused by MPTP intoxication (Fig. 7c). At the level of the SN, MPTP induced a ~35% of SNpc dopaminergic neuronal loss, as determined by stereological cell counts of SNpc TH-positive cells (Fig. 7d). Again, TOM20 overexpression did not prevent dopaminergic cell loss (Fig. 7d). In fact, TOM20 overexpression induced even further dopaminergic cell death compared to sham animals (Fig. 7d). These results indicate that TOM20 overexpression cannot attenuate complex I-induced cell death in the context of the whole organism.

**Fig. 8:**
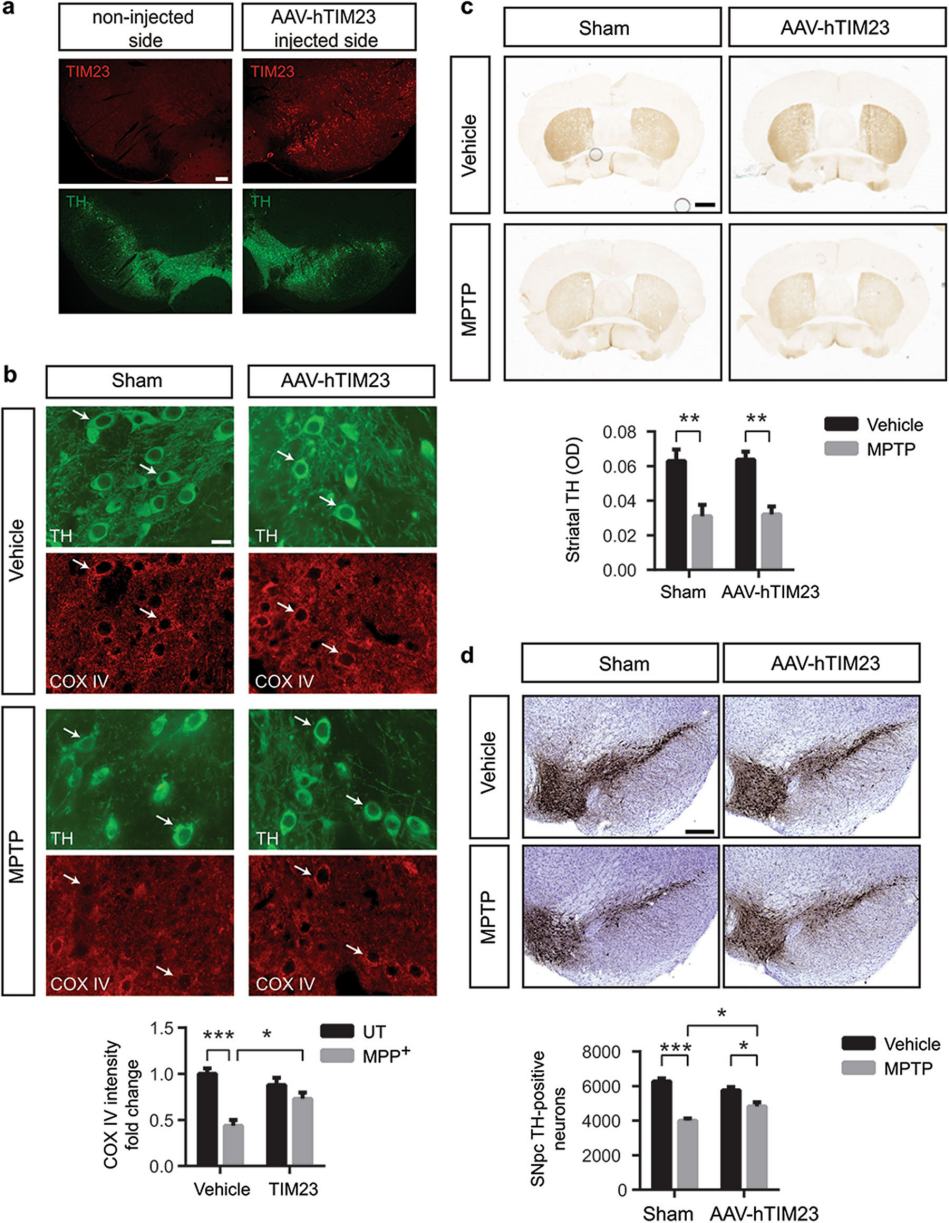
TIM23 overexpression partialy attenuates MPTP-induced dopaminergic neuron injury in vivo. **a** Representative images of TIM23 and TH immunofluorescence in ventral midbrain sections from AAV-hTIM23 mice (*n* = 3). Scale bar: 150 μm. **b**–**d** Sham- and AAV-hTIM23-injected mice were treated with vehicle or MPTP. **b** Representative images of TH and COX IV immunofluorescence in ventral midbrain sections (Sham-Vehicle *n* = 5; Sham-MPTP *n* = 6; AAV-hTIM23-Vehicle *n* = 5; AAV-hTIM23-MPTP *n* = 6). Quantification is depicted as the fold change in COX IV intensity compared with sham-injected vehicle-treated group (*P < 0.05, ***P < 0.001 after two-way ANOVA followed by Tukey’s post hoc test). Scale bar: 50 μm. **c** Representative photomicrographs of TH immunohistochemistry in striatum (Sham-Vehicle *n* = 7; Sham-MPTP *n* = 5; AAV-hTIM23-Vehicle *n* = 8; AAV-hTIM23-MPTP *n* = 8). Quantification is depicted as the optical densitometry of striatal TH immunoreactivity in the different experimental groups at day 21 post MPTP (**P < 0.01 after two-way ANOVA followed by Tukey’s post hoc test). Scale bar: 500 μm. **d** Representative photomicrographs of TH immunohistochemistry SNpc (Sham-Vehicle *n* = 14; Sham-MPTP *n* = 10; AAV-hTIM23-Vehicle *n* = 7; AAV-hTIM23-MPTP *n* = 8). Quantification is depicted as the stereological cell counts of SNpc TH-immunoreactive neurons in the different experimental groups at day 21 post MPTP (*P < 0.05, ***P < 0.001 after two-way ANOVA followed by Tukey’s post hoc test). Scale bar: 500 μm. Error bars indicate s.e.m.

We next focused on whether TIM23 overexpression in vivo is able to mitigate the loss in the mitochondrial protein import and the neuronal cell death observed in the MPTP mouse model. Again, TIM23 was overexpressed unilaterally in the SNpc by means of AAV vectors leading to a 53% ± 2.92 transduction efficiency (Fig. 8a). MPTP intoxication induced a reduction in mitochondrial protein import as determined by the decrease in the mitochondrially localized COX IV protein, which was reversed by TIM23 overexpression (Fig. 8b), indicating that TIM23 overexpression can also rescue mitochondrial protein import in vivo in terms of COX IV immunofluorescence levels. While TIM23 overexpression did not prevent the loss of striatal terminals induced by MPTP (Fig. 8c), it attenuated MPTP-induced nigrostriatal dopaminergic denervation at the level of SNpc dopaminergic cell bodies (Fig. 8d). All together, these data point at an impairment of the mitochondrial protein import system as another pathological factor contributing to the mitochondrial demise and neurodegeneration in the context of PD.

## Discussion

Mitochondrial protein import was previously described to be impaired by α-synuclein in the context of PD^14–16^ but little was known concerning the mechanisms leading to impaired mitochondrial protein import in a context of mitochondrial dysfunction independent of α-synuclein. Here we have followed a combined strategy in order to analyze the mitochondrial protein import machinery in in vitro and in vivo models of PD. We have used complementary techniques (assays of mitochondrial protein import, levels of endogenous nuclear-encoded mitochondrial proteins, translocases protein levels) previously used in other PD-related mouse and cellular models^16^ in order to provide strong evidence of the mitochondrial protein import defect present upon complex I inhibition. Decreases in the mitochondrial protein import activity were linked to reduced protein levels of various OXPHOS subunits, both nuclear-encoded and mitochondrial-encoded. Interestingly enough, exposure to a low dose of MPP^+^ sustained over time specifically impairs mitochondrial translation activity in cells^34^. We think that, in our model, MPP^+^ is exerting a double detrimental effect; on one side it disrupts the mitochondrial protein import, hampering the entry of nuclear-encoded mitochondrial proteins, and, on the other side, it downregulates the translation of mitochondrial-encoded proteins. However, we cannot rule out that the downregulation of OXPHOS proteins is also consequence of other MPP^+^-dependent mechanisms. It is known that downregulation of matrix mitochondrial protein import could represent a secondary event after loss of mitochondrial membrane potential^4^. The membrane potential plays a dual role in the import process through the inner membrane; it activates the channel protein itself^8^ and exerts an electrophoretic effect on the positively charged presequences to drive the presequences to the matrix side^35^. Inner mitochondrial membrane protein import requires the membrane potential and, vice versa, the membrane potential is generated by the proton pumping complexes of the respiratory chain (specifically from complex I, complex III and complex IV) whose formation relies on mitochondrial protein import. We and others have shown that inhibition of complex I activity disrupts the membrane potential^36,37^. Here we have shown that the levels of the mitochondrial protein import proteins, TOM20 and TIM23, are decreased after complex I inhibition. Therefore, both phenomena are certainly inter-connected and acting on one or the other might improve mitochondrial function. Interestingly enough, overexpression of TOM20 partially rescues mitochondrial membrane potential, indicating that improving mitochondrial protein import could potentially have an effect on the mitochondrial membrane potential.

To our knowledge, this is the first time that components of the mitochondrial protein import system are modulated as a potential target to prevent neurodegeneration in PD models. Previous studies have focused on the mechanism by which α-synuclein impairs import^16^ or have used models in which there is no overt neurodegeneration^14^. Knockdown of TOM40 or TIM23 in primary cortical and striatal neurons caused increased cell death^38^, reinforcing the notion that mitochondrial protein import is essential for neuronal survival. Therefore, our studies have also focused on in vivo modulation of mitochondrial protein import system on PD context. In contrast to the in vitro results, TOM20 overexpression in vivo did not restore mitochondrial protein import activity nor protect against MPTP-induced neurodegeneration but rather increased cell death. Moreover, upon transient TOM20 overexpression but not TIM23, mitochondria appeared hyperfused and clustered around the nucleus. This effect of TOM20 on mitochondrial morphology was already reported by Yano et al.^39^. Indeed, aggregation and perinuclear clustering of mitochondria has been observed in relation to some forms of apoptotic cell death^40–42^, upon Bax^43^, Bim^44^ and Bid^45^ induction and during the mitophagy process upon Parkin translocation to the mitochondria^46^. Thus, we hypothesize that the perinuclear mitochondrial clustering correlates with the increased cell death observed upon TOM20 overexpression. However, the exact mechanism by which TOM20 overexpression in vivo induces cell death is unclear.

In contrast, TIM23 delivery in vivo restored mitochondrial protein import and exerted a slight degree of neuroprotection. In fact, TIM23 interacts with some subunits of the respiratory chain and this can be particularly important for the insertion of inner membrane proteins in situations where mitochondrial membrane potential is reduced^47^. Therefore, it is possible that the potential neuroprotective role of TIM23 through increases in the rate of protein import into mitochondria might also impact on the stabilization of complex I subunits. Indeed, TIM23 important role in mitochondrial physiology is illustrated by the fact that it is essential in mice^48^ and yeast^49^. No mutations in humans have been described yet, suggesting that they might lead to embryonic lethality. The first indication that defects in mitochondrial protein import could lead to disease was demonstrated by human deafness dystonia syndrome, caused by mutation in deafness dystonia peptide 1 (DDP1), the human homolog of TIM8, leading to an impaired DDP1–hTIM13 association^50,51^. Interestingly, DDP1 and hTIM13 cooperate together in the import of TIM23 into the mitochondria^52^. Notwithstanding, the majority of the diseases caused by alterations in proteins related with mitochondrial protein import mostly affect high energetic tissues, such as brain or muscle, indicating that this process plays an essential role in the maintenance of cells that heavily rely on mitochondrial function for survival.

In summary, our results have identified the disruption of mitochondrial protein import machinery as a pathogenic event in the context of PD. Even though overexpression of single subunits of the translocases system has not been enough to fight against neurodegeneration, we strongly think that a strategy aimed to target several key elements in the pathway could have a major impact. Thus, identification of potential drugs/compounds able to boost mitochondrial protein import activity could represent a potential therapeutic strategy in the field of PD.

## Electronic supplementary material

Supplementary Figure 1

Supplementary Figure 2

Supplementary Figure 3

Supplementary Figure 4

Supplementary Figure 5

Supplementary figure legends

## References

[1] Franco-Iborra S, Vila M, Perier C (2016). The Parkinson disease mitochondrial hypothesis. Neurosci.

[2] Schapira AH (1990). Mitochondrial complex I deficiency in Parkinson’s disease. J. Neurochem..

[3] Anderson S (1981). Sequence and organization of the human mitochondrial genome. Nature.

[4] Wiedemann N, Pfanner N (2017). Mitochondrial machineries for protein import and assembly. Annu. Rev. Biochem..

[5] Kiebler M (1990). Identification of a mitochondrial receptor complex required for recognition and membrane insertion of precursor proteins. Nature.

[6] Shiota T (2015). Molecular architecture of the active mitochondrial protein gate. Science.

[7] Schmidt O, Pfanner N, Meisinger C (2010). Mitochondrial protein import: from proteomics to functional mechanisms. Nat. Rev. Mol. Cell Biol..

[8] Truscott KN (2001). A presequence- and voltage-sensitive channel of the mitochondrial preprotein translocase formed by Tim23. Nat. Struct. Biol..

[9] Wasilewski M, Chojnacka K, Chacinska A (2017). Protein trafficking at the crossroads to mitochondria. Biochim. Biophys. Acta.

[10] Tranebjaerg L (1995). A new X linked recessive deafness syndrome with blindness, dystonia, fractures, and mental deficiency is linked to Xq22. J. Med. Genet..

[11] Davey KM (2005). Mutation of DNAJC19, a human homologue of yeast inner mitochondrial membrane co-chaperones, causes DCMA syndrome, a novel autosomal recessive Barth syndrome-like condition. J. Med. Genet..

[12] Ghiglieri V, Calabrese V, Calabresi P (2018). Alpha-Synuclein: from early synaptic dysfunction to neurodegeneration. Front. Neurol..

[13] Nakamura K (2013). α-Synuclein and mitochondria: partners in crime?. Neurotherapeutics.

[14] Bender A (2013). TOM40 mediates mitochondrial dysfunction induced by alpha-synuclein accumulation in Parkinson’s disease. PLoS One.

[15] Devi L, Raghavendran V, Prabhu BM, Avadhani NG, Anandatheerthavarada HK (2008). Mitochondrial import and accumulation of alpha-synuclein impair complex I in human dopaminergic neuronal cultures and Parkinson disease brain. J. Biol. Chem..

[16] Di Maio R (2016). α-Synuclein binds TOM20 and inhibits mitochondrial protein import in Parkinson’s disease. Sci. Transl. Med..

[17] Frezza C, Cipolat S, Scorrano L (2007). Organelle isolation: functional mitochondria from mouse liver, muscle and cultured filroblasts. Nat. Protoc..

[18] Moisoi N (2009). Mitochondrial dysfunction triggered by loss of HtrA2 results in the activation of a brain-specific transcriptional stress response. Cell Death Differ..

[19] Mokranjac D, Neupert W (2007). Protein import into isolated mitochondria. Methods Mol. Biol..

[20] Stojanovski D, Bohnert M, Pfanner N, van der Laan M (2012). Mechanisms of protein sorting in mitochondria. Cold Spring Harb. Perspect. Biol..

[21] Larsson NG (2010). Somatic mitochondrial DNA mutations in mammalian aging. Annu. Rev. Biochem..

[22] Haynes CM, Ron D (2010). The mitochondrial UPR - protecting organelle protein homeostasis. J. Cell Sci..

[23] Nakai M, Mori A, Watanabe A, Mitsumoto Y (2003). 1-methyl-4-phenylpyridinium (MPP+) decreases mitochondrial oxidation-reduction (REDOX) activity and membrane potential (Deltapsi(m)) in rat striatum. Exp. Neurol..

[24] Perier C (2005). Complex I deficiency primes Bax-dependent neuronal apoptosis through mitochondrial oxidative damage. Proc. Natl. Acad. Sci. USA.

[25] Ramage L, Junne T, Hahne K, Lithgow T, Schatz G (1993). Functional cooperation of mitochondrial protein import receptors in yeast. EMBO J..

[26] Söllner T, Griffiths G, Pfaller R, Pfanner N, Neupert W (1989). MOM19, an import receptor for mitochondrial precursor proteins. Cell.

[27] Bauer MF, Sirrenberg C, Neupert W, Brunner M (1996). Role of Tim23 as voltage sensor and presequence receptor in protein import into mitochondria. Cell.

[28] Nicklas WJ, Vyas I, Heikkila RE (1985). Inhibition of NADH-linked oxidation in brain mitochondria by 1-methyl-4-phenyl-pyridine, a metabolite of the neurotoxin, 1-methyl-4-phenyl-1,2,5,6-tetrahydropyridine. Life. Sci..

[29] Hasegawa E, Takeshige K, Oishi T, Murai Y, Minakami S (1990). 1-Methyl-4-phenylpyridinium (MPP+) induces NADH-dependent superoxide formation and enhances NADH-dependent lipid peroxidation in bovine heart submitochondrial particles. Biochem. Biophys. Res. Commun..

[30] Rossetti ZL, Sotgiu A, Sharp DE, Hadjiconstantinou M, Neff NH (1988). 1-Methyl-4-phenyl-1,2,3,6-tetrahydropyridine (MPTP) and free radicals in vitro. Biochem. Pharmacol..

[31] Ramonet D (2013). Optic atrophy 1 mediates mitochondria remodeling and dopaminergic neurodegeneration linked to complex I deficiency. Cell Death Differ..

[32] Perier C (2007). Two molecular pathways initiate mitochondria-dependent dopaminergic neurodegeneration in experimental Parkinson’s disease. Proc. Natl. Acad. Sci. USA.

[33] Vila M (2001). Bax ablation prevents dopaminergic neurodegeneration in the 1-methyl- 4-phenyl-1,2,3,6-tetrahydropyridine mouse model of Parkinson’s disease. Proc. Natl. Acad. Sci. USA.

[34] Zhu JH (2012). Impaired mitochondrial biogenesis contributes to depletion of functional mitochondria in chronic MPP+toxicity: dual roles for ERK1/2. Cell Death Dis..

[35] Martin J, Mahlke K, Pfanner N (1991). Role of an energized inner membrane in mitochondrial protein import. Delta psi drives the movement of presequences. J. Biol. Chem..

[36] Perier C, Vila M (2012). Mitochondrial biology and Parkinson’s Disease. Cold Spring Harb. Perspect. Med..

[37] Perier C (2013). Accumulation of mitochondrial DNA deletions within dopaminergic neurons triggers neuroprotective mechanisms. Brain.

[38] Yano H (2014). Inhibition of mitochondrial protein import by mutant huntingtin. Nat. Neurosci..

[39] Yano M (1997). Visualization of mitochondrial protein import in cultured mammalian cells with green fluorescent protein and effects of overexpression of the human import receptor Tom20. J. Biol. Chem..

[40] De Vos K (1998). The 55-kDa tumor necrosis factor receptor induces clustering of mitochondria through its membrane-proximal region. J. Biol. Chem..

[41] Suen YK (2000). Concanavalin A induced apoptosis in murine macrophage PU5-1.8 cells through clustering of mitochondria and release of cytochrome c. Apoptosis.

[42] Yee KS, Wilkinson S, James J, Ryan KM, Vousden KH (2009). PUMA- and Bax-induced autophagy contributes to apoptosis. Cell Death Differ..

[43] Desagher S, Martinou JC (2000). Mitochondria as the central control point of apoptosis. Trends Cell Biol..

[44] Puthalakath H, Huang DC, O’Reilly LA, King SM, Strasser A (1999). The proapoptotic activity of the Bcl-2 family member Bim is regulated by interaction with the dynein motor complex. Mol. Cell.

[45] Li H, Zhu H, Xu C, Yuan J (1998). Cleavage of BID by caspase 8 mediates the mitochondrial damage in the Fas pathway of apoptosis. Cell.

[46] Okatsu K (2010). p62/SQSTM1 cooperates with Parkin for perinuclear clustering of depolarized mitochondria. Genes. Cells.

[47] Wiedemann N, van der Laan M, Hutu DP, Rehling P, Pfanner N (2007). Sorting switch of mitochondrial presequence translocase involves coupling of motor module to respiratory chain. J. Cell Biol..

[48] Ahting U (2009). Neurological phenotype and reduced lifespan in heterozygous Tim23 knockout mice, the first mouse model of defective mitochondrial import. Biochim. Biophys. Acta.

[49] Maarse AC, Blom J, Keil P, Pfanner N, Meijer M (1994). Identification of the essential yeast protein MIM17, an integral mitochondrial inner membrane protein involved in protein import. FEBS Lett..

[50] Jin H (1996). A novel X–linked gene, DDP, shows mutations in families with deafness (DFN–1), dystonia, mental deficiency and blindness. Nat. Genet..

[51] Roesch K, Curran SP, Tranebjaerg L, Koehler CM (2002). Human deafness dystonia syndrome is caused by a defect in assembly of the DDP1/TIMM8a-TIMM13 complex. Hum. Mol. Genet..

[52] Rothbauer U (2001). Role of the deafness dystonia peptide 1 (DDP1) in import of human Tim23 into the inner membrane of mitochondria. J. Biol. Chem..

