# Supplementary material for: Defective mitochondrial protein import contributes to complex I-induced mitochondrial dysfunction and neurodegeneration in Parkinson’s disease
Source: Cell Death Dis. 2018 Nov 7;9(11):1122. doi: 10.1038/s41419-018-1154-0 (PMC6221944; doi:10.1038/s41419-018-1154-0)
Supplement: Supplementary file 6 — Supplementary figure legends [file 41419_2018_1154_MOESM6_ESM.docx]

**Supplementary Figure Legend :**

**Supplementary Figure 1**

Quantification of mitochondrial DNA (mtDNA) copy number in BE(2)-M17 cells UT or treated with MPP^+^ (0.25 mM, 0.5 mM and 1 mM, 24h, n=3 independent experiments). mtDNA copy number was measured by quantitative RT-PCR and expressed as the ratio of mtDNA (*12S* copy number) to nuclear DNA (*RNAseP* copy number). Quantification is depicted as fold change to UT condition. One-way ANOVA test followed by Tukey’s post hoc test. Error bars indicate s.e.m.

**Supplementary Figure 2**

**(a)** Representative immunoblots of TOM20 and VDAC protein levels in BE(2)-M17 cells untransfected (Veh) or pCMV6-XL5-TOM20-transfected (TOM20) during 24h or 48h. Protein levels were normalized relative to VDAC (n=3 independent experiments). Quantification is depicted as fold change to Veh condition (* P<0.05, ** P< 0.01 after one way ANOVA test followed by Tukey’s post hoc test). Error bars indicate s.e.m. **(b)** Representative images of TOM20 and Mitotracker Deep Red (MT-DR) immunostained in vehicle- or pCMV6-XL5-TOM20-transfected (TOM20) BE(2)-M17 cells.

**Supplementary Figure 3**

**(a)** Representative immunoblots of TIM23 and VDAC protein levels in BE(2)-M17 cells untransfected (Veh) or pCMV6-XL5-TIM23-transfected (TIM23) during 24h or 48h. Protein levels were normalized relative to VDAC (n=3 independent experiments). Quantification is depicted as fold change to Veh condition (*** P<0.001 after one way ANOVA test followed by Tukey’s post hoc test). Error bars indicate s.e.m. **(b)** Representative images of TIM23 and Mitotracker Deep Red (MT-DR) immunostained in vehicle- or pCMV6-XL5-TIM23-transfected (TIM23) BE(2)-M17 cells.

**Suplementary Figure 4**

Representative images of TIM23 and TOM20 immunohistochemistry in ventral midbrain sections from vehicle- and MPTP-treated mice euthanized at day 2 after the last injection. Dashed lines enclose substantia nigra. Scale bar: 500 μm.

**Supplementary Figure 5**

Quantification of mitochondrial DNA (mtDNA) copy number in ventral midbrain samples of vehicle- (n=6) and MPTP-treated (n=7-8) mice euthanized at different time points. mtDNA copy number was measured by quantitative RT-PCR and expressed as the ratio of mtDNA (*16S* and *ND4* copy number) to nuclear DNA (*ANG1* copy number) (* P<0.05, ** P<0.01, *** P<0.001 after one-way ANOVA followed by Tukey’s post hoc test). Error bars indicate s.e.m.
